# Prevalence of Acute Coronary Syndrome and Various Risk Factors in Acute Stroke Patients

**DOI:** 10.7759/cureus.9552

**Published:** 2020-08-04

**Authors:** Muhammad Humayoun Rashid, Ghulam Yaseen, Umar Ghaffar, Ahmad Ali Khan, Ahmad Kabir, Azka Aisha, Aqsa Komel

**Affiliations:** 1 Internal Medicine, Nishtar Medical University, Multan, PAK; 2 Cardiology, Ch. Pervaiz Elahi Institute of Cardiology, Multan, PAK; 3 Pathology, Ch. Pervaiz Elahi Institute of Cardiology, Multan, PAK; 4 Anesthesia & Critical Care, Ch. Pervaiz Elahi Institute of Cardiology, Multan, PAK

**Keywords:** acute coronary syndrome, stroke, hypertension, dyslipidemia, diabetes mellitus, smoking, obesity

## Abstract

Background

Stroke is the second leading cause of death worldwide after acute coronary syndrome (ACS). Both diseases share many risk factors such as hypertension, diabetes, dyslipidemia, and smoking. Patients who experience acute coronary syndrome are at heightened risk of recurrent ischemic events such as ischemic strokes, one of the most feared cardiovascular events because of the risk of long-term disability. We tried to estimate the prevalence of underlying ACS among patients with acute stroke.

Methods

This cross-sectional study was done at the CPE Institute of Cardiology, Pakistan, and Nishtar Medical University and Hospital, Pakistan. A total of 160 acute stroke cases were selected by consecutive sampling technique and questionnaire forms were filled. Detailed history, investigations, and physical examinations were done. The primary outcome was the prevalence of ACS and secondary outcomes were the prevalence of hypertension, smoking, dyslipidemia, diabetes mellitus, and previous history of stroke in stroke patients.

Results

Most of the patients that presented to us were above 50 years of age with the mean age of 62 years (SD = 9.23 years). Male predominance was seen with a total of 115 (72%) cases. Out of 160 patients, 91 (57%, p < 0.05) had underlying ACS, with 45 cases (49%) with unstable angina, 20 (22%) with non-ST-elevation myocardial infarction (NSTEMI), and 26 (29%) with ST-elevation myocardial infarction (STEMI). Prevalence of risk factors in 160 cases were, hypertension (101, 63%), lack of exercise (91, 57%), smoking (70, 44%), diabetes mellitus (61, 38%), dyslipidemia (50, 31%). All these results were statistically significant (p < 0.05). Prevalence of obesity (35, 22%) and previous stroke history (19, 12%) was statistically insignificant (p > 0.05).

Conclusion

Acute coronary syndrome is still frequently present in patients with acute stroke. The need of the hour is to manage ACS efficiently so that its deadly complications such as stroke can be prevented.

## Introduction

Stroke is the second leading cause of death worldwide after acute coronary syndrome (ACS). Both diseases share a lot of risk factors such as hypertension, diabetes, dyslipidemia, and smoking. ACS is diagnosed when patients present with unstable angina, non-ST-elevation myocardial infarction (NSTEMI), or ST-elevation myocardial infarction (STEMI). Such patients have a wide spectrum of risks for death and cardiovascular ischemic events. Careful risk assessment of ACS patients helps clinicians to determine prognosis and may, therefore, be useful in guiding management and providing valuable information to patients [1,2]. To be clinically practical, a risk stratification model must be straightforward and use clinical risk factors that are readily ascertainable at hospital presentation.

ACS and some subtypes of ischemic stroke share similar pathophysiology, including inflammation and the development of atherosclerosis. The acute coronary syndrome can itself be a risk factor for stroke due to thromboembolism, or ischemia-induced atrial fibrillation, or stasis of blood. Several scoring methods, including GRACE (Global Registry of Acute Coronary Events) [3], TIMI (Thrombolysis in Myocardial Infarction) [4], and PURSUIT (Platelet glycoprotein IIb/IIIa in Unstable angina: Receptor Suppression Using Integrilin Therapy) [5], are developed to assess ACS patients who are at risk of developing complications so that they can be managed efficiently and timely to reduce disability. Stroke is a rare but serious complication of acute coronary syndrome. At present, no specific score exists to identify patients at higher risk of developing stroke after ACS. The present study aims to check how many of the stroke patients presenting in the emergency and outpatient department have the underlying acute coronary syndrome.

## Materials and methods

Study design

This cross-sectional study took place at the CPE Institute of Cardiology Multan, Pakistan, and Nishtar Medical University and Hospital Multan, Pakistan. Ethics committee approval was obtained from both the hospitals. The non-probability consecutive sampling technique was used. A sample of 160 acute stroke cases, from January 2020 to June 2020, presenting in emergency, outpatient and inpatient departments, was taken on board to fill a questionnaire form. Inclusion criteria were age 30-80, either gender and diagnosed stroke either hemorrhagic or ischemic. Exclusion criteria were patients with age less than 30 years or greater than 80 years, patients with chronic obstructive pulmonary disease, coagulation disorders, antiphospholipid syndrome, HIV, recent surgical operations, pregnancy, and malignancy.

American Heart Association definitions for ACS subtypes were used. Unstable angina is defined as anginal pain that occurs in a resting state with neither ST-segment elevation, nor elevated biomarkers of myocardial ischemia. NSTEMI is defined as chest pain with elevated biomarkers of myocardial ischemia (cardiac enzymes, troponins) but no ST-segment elevations. STEMI is defined as chest pain with elevated biomarkers of myocardial ischemia and ST-segment elevations or new-onset left bundle branch block. Stroke was defined as neurological dysfunction caused by an ischemic or hemorrhagic event with residual symptoms at least 24 h after onset or leading to death [6].

Outcomes

The primary outcome of our study was to know the prevalence of acute coronary syndrome in stroke patients on retrospective analysis, and which type of acute coronary syndrome was most prevalent among them. As our secondary outcomes, characteristics of the patients such as age, gender, diabetes mellitus, hypertension, dyslipidemia, smoking, and previous stroke episode were noted down and their prevalence in stroke patients was taken into consideration.

Data collection

A total of 160 stroke patients that met our inclusion criteria were selected by a convenience sampling method using consecutive sampling technique and they were properly educated about the study. Informed consent was taken from all of them. Stroke type was diagnosed mostly by brain computed tomography (CT) scan or magnetic resonance imaging to detect either infarction or hemorrhage (intracerebral, subdural, or subarachnoid). A detailed history and physical examinations were done. Each patient underwent a series of investigations including CT scan brain, ECG, transthoracic echocardiography, HbA1C, lipid profile, and carotid Doppler studies. Their previous records of the acute coronary syndrome were checked thoroughly. Information regarding the use of ACS medication, any ACS intervention that has been done previously, hospital admissions, previous ECGs, cardiac biomarker reports, and echocardiography reports, was taken. Our team collaborated and divided the tasks of history taking, examination, following the investigations, and gathering data of their previous hospital registrations.

Data analysis

Data was entered and analyzed in Microsoft Office Excel 2016 and SPSS Statistics version 19 (IBM Corp., Armonk, NY). Mean and standard deviations were calculated for continuous variables such as age, and the student’s t-test was used for analysis. Frequencies and percentages were calculated for categorical variables such as ACS, diabetes mellitus, hypertension, gender, dyslipidemia, smoking, and previous stroke or transient ischemic attack (TIA). The Chi-square test was used for analysis. All the data was compiled in the form of flow charts, spreadsheets, and histograms.

## Results

**Figure 1:**
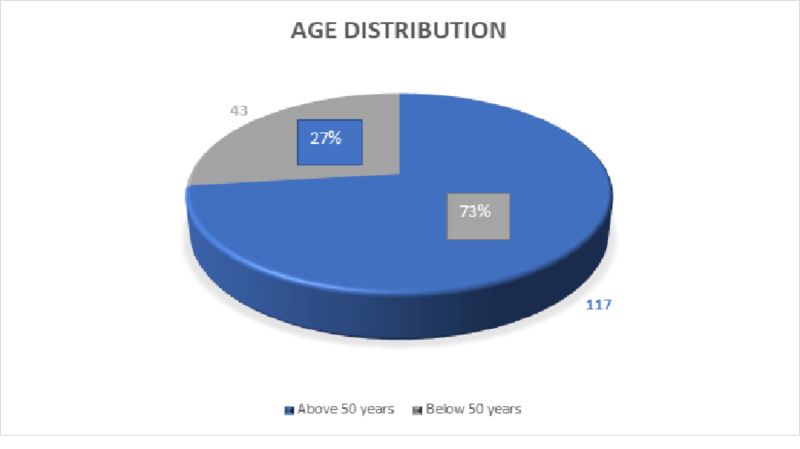
Age distribution pie chart

The mean age of the patients was 62 years ± 9.23 years (standard deviation). The minimum age of stroke presentation was 34 years and the maximum age of presentation was 78 years as shown in Table 1. Out of 160 cases that were recruited in the study, 117 (73%) were above 50 years of age and 43 (27%) were under the age of 50 years at the time of presentation as shown in Figure 1.

**Table 1:** Age distribution SD: Standard deviation

	MINIMUM	MAXIMUM	MEAN	SD
AGE	34	78	62	± 9.23 years

**Figure 2:**
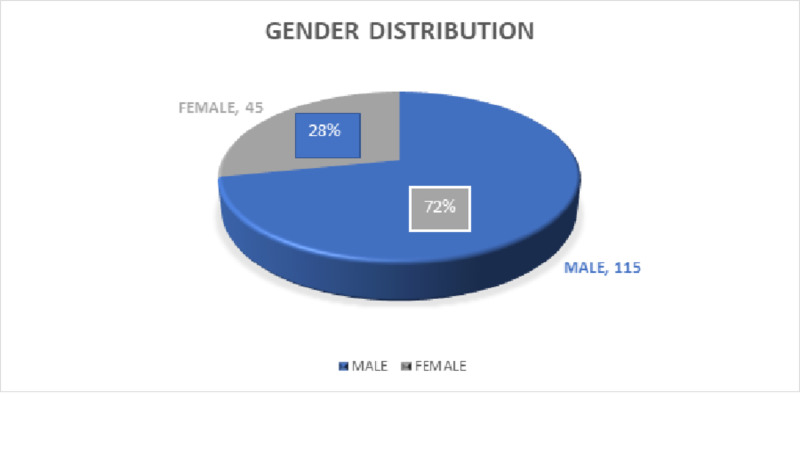
Gender distribution pie chart

Non-probability consecutive sampling technique was followed. Out of 160 cases, 115 (72%) male patients, and 45 (28%) female patients presented to us. This showed male predominance in stroke cases concluding that male gender itself possesses more risk for stroke occurrence. The results are displayed in Figure 2.

**Figure 3:**
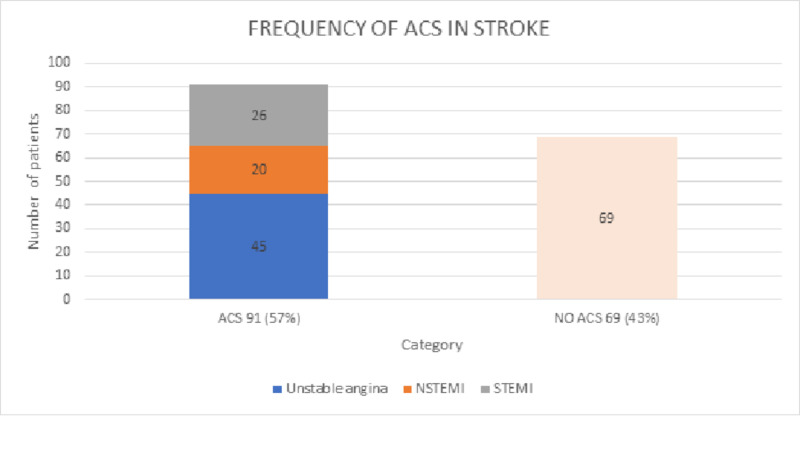
Prevalence of ACS in acute stroke ACS: Acute coronary syndrome; NSTEMI: Non-ST-elevation myocardial infarction; STEMI: ST-elevation myocardial infarction.

As our primary outcome we analyzed that out of 160 cases, 91 cases i.e. 57% (p < 0.05) had underlying acute coronary syndrome associated with stroke, and 69 cases i.e. 43% (p < 0.05) had no underlying acute coronary syndrome in an association. Among the 91 ACS cases, 45 (49%) had unstable angina, 20 (22%) had NSTEMI, and 26 (29%) had STEMI. The data is compiled in Table 2 and presented graphically in Figure 3.

**Table 2:** Prevalence of ACS in acute stroke ACS: Acute coronary syndrome; NSTEMI: Non-ST-elevation myocardial infarction; STEMI: ST-elevation myocardial infarction.

STROKE PATIENTS	NO ACS		ACS	
(n = 160)		UNSTABLE ANGINA	NSTEMI	STEMI
Number	69	45	20	26
Percentage	43%	49%	22%	29%

**Figure 4:**
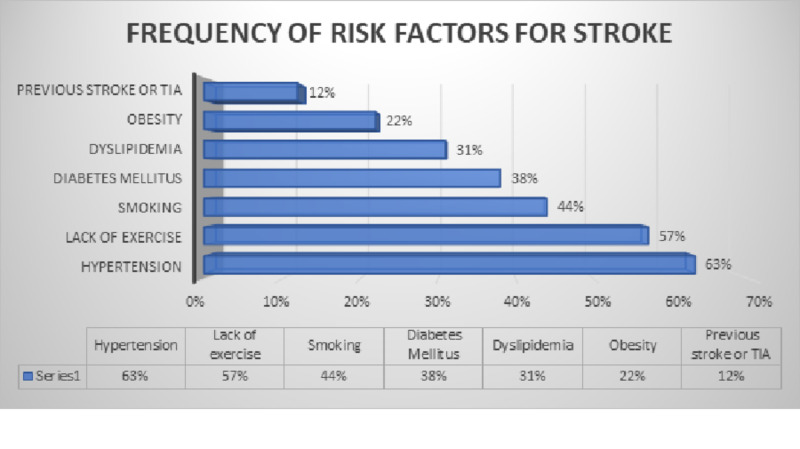
Risk factors frequencies in stroke patients TIA: Transient ischemic attack

We studied the prevalence of risk factors that were present in the stroke patients that were included in the study. Questionnaires of our study included questions about the risk factors that are associated with stroke according to the American Academy of Neurology and the American Heart Association and were filled by the attendants of the patients mostly. This showed that among 160 stroke patients, 101 (63%) had hypertension, 91 (57%) had lack of exercise, 70 (44%) had smoking history, 61 (38%) had diabetes mellitus, 50 (31%) had dyslipidemia, 35 (22%) were obese and 19 (12%) had the previous history of stroke or TIA. These results are displayed in the following Figure 4.

Stratification of hypertension, lack of exercise, smoking, diabetes mellitus, dyslipidemia, obesity, and previous stroke or TIA with stroke, and acute coronary syndrome is given in Table 3. This table shows that hypertension is strongly associated with both stroke and ACS with 63% and 55% (p < 0.05) occurrence respectively and so do lack of exercise with 57% and 69% (p < 0.05), respectively.

**Table 3:** Prevalence of various risk factors in acute stroke TIA: Transient ischemic attack

RISK FACTORS	STATUS	STROKE PATIENTS (160)	PERCENTAGE	ACS PATIENTS (91)	PERCENTAGE	P-VALUE
Hypertension	Present	101	63%	50	55%	<0.05
	Absent	59	37%	41	45%	
Lack of exercise	Present	91	57%	63	69%	<0.05
	Absent	69	43%	28	31%	
Smoking	Present	70	44%	32	35%	<0.05
	Absent	90	56%	59	65%	
Diabetes mellitus	Present	61	38%	36	40%	<0.05
	Absent	99	62%	55	60%	
Dyslipidemia	Present	50	31%	25	27%	<0.05
	Absent	110	69%	66	73%	
Obesity	Present	35	22%	16	18%	>0.05
	Absent	125	78%	75	82%	
Previous stroke or TIA	Present	19	12%	6	7%	>0.05
	Absent	141	88%	85	93%	

## Discussion

We did a cross-sectional study to estimate the prevalence of acute coronary syndrome in patients with acute stroke onset. Patients who experience acute coronary syndrome are at heightened risk of recurrent ischemic events [7-9]. These events are not limited to the coronary vascular bed but also include ischemic strokes, one of the most feared cardiovascular events because of the risk of long-term disability [10]. Hence, we tried to estimate the prevalence of underlying ACS among patients with acute stroke and the result came out to be 57% (p < 0.05) and was statistically significant. It shows that ACS is still very much prevalent in stroke. Apart from the fact that the pathophysiology and most of the risk factors for stroke and ACS are similar, ACS itself is a risk factor for ischemic stroke mostly due to stasis of blood in ventricles, ACS induced atrial fibrillation and turbulence of blood can cause clot formation in chambers of the heart which can dislodge and travel through major arteries towards brain causing a stroke. Studies also indicate that myocardial infarction is a major risk factor for acute stroke onset [11,12]. The efficient management of acute coronary syndrome is required so that such deadly complications like a stroke can be reduced.

Most of the cases that presented to us were above the age of 50 years. And the mean age came out to be 62 years. It is explained partly by the fact that with age the vessels become less elastic and more fragile, and also that the aged people have increased number of co-morbidities like hypertension, diabetes, and dyslipidemias. Similarly, the male gender was found to have a higher incidence of stroke with up to the four-fold increased risk of stroke. Hence age and gender are uncontrollable risk factors and independent predictors of acute stroke onset. Most of the risk factors identified in this study correlated with those that have been identified in other studies. Risk factors that have appeared repeatedly in other studies include older age, history of a previous stroke, hypertension, diabetes mellitus, dyslipidemia, carotid and peripheral vascular disease, obesity, and atrial fibrillation. It is interesting to note that most of the risk factors for stroke in the cases we studied, closely mirror those contained in the CHA2DS2-VASc scoring system, which was derived and validated as a tool for stroke risk stratification in patients with atrial fibrillation [13-15]. Atrial fibrillation (AF) is associated with a ≈5-fold increased risk of ischemic stroke, and stroke prevention is a major priority in the clinical management of AF. When compared with control/placebo, oral anticoagulation (OAC) therapy reduces the risk of stroke by 64%, and the risk of death by 26% [16]. A systematic review and meta-analysis on CHA2DS2-VASc risk factors as predictors of stroke after acute coronary syndrome also studied that they have statistically significant predictive value for stroke onset in the patients having underlying ACS [17].

Our study indicates that both hypertension and diabetes mellitus are common in patients with stroke and ACS. Hypertension was 63% prevalent in stroke patients and 55% in ACS patients. Similarly, diabetes mellitus was 38% prevalent in stroke and 40% in ACS patients. These risk factors cause atherosclerosis and arteriosclerosis which can lead to turbulence of blood flow, plaque formation, and rupture leading to thrombus formation and ischemic events, commonly in heart and brain [18-21]. Dyslipidemia is also found commonly in stroke and ACS patients. Our study showed 31% and 27% prevalence, respectively. A large randomized controlled trial on 3216 patients showed that adults with both high triglyceride (TG) and low high-density lipoprotein cholesterol (HDL-C), particularly those with diabetes, have increased risks of coronary heart disease and stroke. In particular, those with an LDL-C level of ≥130 mg/dL may have an increased risk of incident stroke [22]. Lipid-lowering therapy with statins has been shown to result in a 22% reduction in major vascular events per 1 mmol/L reduction in low-density lipoprotein cholesterol (LDL-C), including a 21% relative risk reduction in ischemic stroke per 1 mmol/L reduction in LDL-C.

Cigarette smoking is an important risk factor for all-cause mortality as well as vascular disease mortality [23,24]. Many prospective cohort studies conducted in Western populations, as well as Asian populations, have indicated a strong and independent relationship between cigarette smoking and risk of stroke [25-28]. Our study also showed a high prevalence of smoking in stroke patients which is 44%. The prevalence of obesity and previous stroke history came out to be 22% and 12% and the results were statistically insignificant. Maybe because the study sample was not high enough to prove the association. Whereas, in an analysis adjusted for age, sex, race, and smoking, obesity was associated with a 65% increased risk for ischemic stroke (odds ratio, 1.65; 95% confidence interval, 1.33-2.04). Some people believe that treating obesity decreases the risk of stroke, while some say that treating the consequences of obesity such as diabetes and hypertension imparts the benefits [29]. These risk factors are still debatable and highly researched topics in cardiology and neurology.

## Conclusions

Acute coronary syndrome is still frequently present in patients with acute stroke. Older age and male gender are independent predictors of stroke occurrence. Risk factors such as hypertension, dyslipidemia, lack of exercise, smoking, and diabetes mellitus are also increasingly prevalent. The need of the hour is to manage ACS efficiently so that its deadly complications such as stroke can be prevented from occurring.
